# Serum concentrations of selective serotonin reuptake inhibitors and atypical antipsychotics in children and adolescents: data from routine clinical care

**DOI:** 10.1007/s00702-025-03021-y

**Published:** 2025-10-09

**Authors:** Sophie Wiegand, Xenia M. Hart, Karin M. Egberts, Hans-Willi Clement, Christian Fleischhaker

**Affiliations:** 1 Department of Child and Adolescent Psychiatry and Psychotherapy, University Medical Center Freiburg, Freiburg, Germany; 2 Department of Molecular Neuroimaging, Medical Faculty Mannheim, Central Institute of Mental Health, University of Heidelberg, Mannheim, Germany; 3 Department of Neuropsychiatry, Keio University School of Medicine, Tokyo, Japan; 4 Department of Child and Adolescent Psychiatry, Psychosomatics and Psychotherapy, Center for Mental Health, University Hospital of Wuerzburg, Wuerzburg, Germany; 5 Department of Child and Adolescent Psychiatry, GGZ Reinier van Arkel, ‘s-Hertogenbosch, The Netherlands

**Keywords:** Selective serotonin reuptake inhibitors, Atypical antipsychotics, Therapeutic reference range, Therapeutic drug monitoring, Children and adolescents

## Abstract

Therapeutic reference ranges (TRRs) for selective serotonin reuptake inhibitors (SSRIs) and atypical antipsychotics are established for adults. In children and adolescents, however, knowledge on TRRs of psychotropic drugs is scarce. The aim of this study was to determine serum concentrations in children and adolescents to provide guidance for the definition of therapeutic reference ranges in this age group. Within the routine therapeutic drug monitoring service of a German child and adolescent psychiatric university hospital, serum concentrations of citalopram, fluoxetine, fluvoxamine, sertraline, aripiprazole, clozapine, olanzapine, quetiapine or risperidone in children and adolescents were collected between 1999 und 2022 and retrospectively analyzed. The 25th to 75th interquartile ranges (IQRs) were calculated and compared with the TRRs defined for adults. In total, 618 serum level concentrations were examined. The IQRs found for fluvoxamine (75–203 ng/ml), sertraline (25–54 ng/ml), olanzapine (13–57 ng/ml), and aripiprazole (93–245 ng/ml) were similar to published therapeutic ranges for adults. Citalopram (18–51 ng/ml), clozapine (180–419 ng/ml), risperidone plus 9-hydroxyrisperidone (11–35 ng/ml), aripiprazole plus dehydroaripiprazole (118–313 ng/ml) and quetiapine (34–234 ng/ml) exhibit somewhat lower ranges in children and adolescents when compared to adults. The IQR found for fluoxetin (102–263 ng/ml) could not be compared with that of adults due to methodological differences. Our results contribute to the knowledge of serum concentrations of SSRIs and atypical antipsychotics in children and adolescents. The differences found in some substances compared to adults clearly indicate that age- and substance-specific therapeutic ranges need to be defined for children and adolescents.

## Introduction

The clinical indications for citalopram, fluoxetine, fluvoxamine and sertraline for adults include the treatment of major depression, recurrence prevention of major depression, obsessive-compulsive disorder (OCD), bulimia, panic disorder, social anxiety disorder and post-traumatic stress disorder (Fachinfo- Service 2022). Fluoxetine is approved by the European Medicines Agency (EMA) for the treatment of depression in children eight years or older (EMA 2006). For OCD, fluvoxamine is authorized from the age of eight and sertraline from the age of six, while citalopram is generally not authorized for this age group (Fachinfo- Service 2022). The effectiveness of SSRIs is based on the improved serotonergic signal transmission in the central nervous system. This occurs through an accumulation of serotonin in the synaptic cleft by selectively blocking the reuptake of serotonin at the presynapse (Said 2015). In adults, the atypical antipsychotics (aripiprazole, clozapine, quetiapine, olanzapine and risperidone) are indicated to treat schizophrenia, aggression in the case of behavioral disorders and below-average intelligence or mental retardation, treatment-resistant schizophrenia, manic and depressive episodes of bipolar disorder and manic episodes. They are also used as a phase prophylaxis or as add-on therapy for depression. In minors, clozapine is approved for treatment-resistant schizophrenia from the age of sixteen. Risperidone can be used temporarily from the age of five to treat behavioral disorders. Aripiprazole is authorized for the treatment of schizophrenia from the age of fifteen and for the treatment of manic episodes in bipolar 1 disorder from the age of thirteen. Quetiapine and olanzapine have not been approved for minors (Fachinfo- Service 2022). The atypical antipsychotics, also known as second-generation antipsychotics, antagonize other receptors in addition to dopamine receptors. Classical antipsychotics or first-generation antipsychotics, on the other hand, mainly antagonize the dopamine D2 receptor (Seifert al. 2022; Ludwig et al.^2022^). Third-generation antipsychotics also belong to the atypical antipsychotics and are partial dopamine agonists; aripiprazole was the first approved drug of this type (Remington 2014). Off-label use of medication is very common in child and adolescent psychiatry and associated with unclear safety and effectiveness (Egberts et al. 2022). One study found that around 96% of antipsychotics and 52% of antidepressants were prescribed off-label. The most common reason was the lack of authorization for the corresponding age (Braüner et al. 2016). The measurement of drug concentrations in the blood during pharmacotherapy in conjunction with their interpretation is referred to as therapeutic drug monitoring (TDM). This method enables individualized pharmacotherapy and is strongly recommended for special patient groups such as elderly, multimorbid patients or children and adolescents. For adults, there are recommendations for concentration ranges of a drug in the blood in which it is probably safe and at the same time therapeutically effective. This is referred to as the therapeutic reference range (TRR). The TRR is usually specified in the literature for the main indication of a drug (Hiemke et al. 2018). A correlation between drug concentration and clinical effect has been demonstrated in fixed-dose studies for several psychoactive drugs. If valid data on the concentration-effect-relationship is available, a TRR can be determined by setting the concentration required for maximum efficacy as the upper limit and the concentration that is more effective than placebo as the lower limit. However, valid studies are not available for many drugs (Hiemke 2019). Various options have been proposed for calculating a preliminary TRR in the absence of valid concentration/ response-studies. In 2004, it was proposed that the mean ± 2 standard deviations (SD) or the 25th to 75th percentile of serum concentrations could serve as a substitute (Bengtsson 2004). In their latest update of the consensus guidelines in neuropsychopharmacology, the TDM Group of the Arbeitsgemeinschaft für Neuropsychopharmakologie und Pharmakopsychiatrie (AGNP) recommended that the mean ± 1 standard deviation of serum concentrations in responders should be calculated (Hiemke et al. 2018). However, the methods that use the mean and standard deviation assume a normal distribution of the data. An analysis comparing these calculations shows that the IQR is the most accurate of three (Hiemke 2019). The AGNP recently prepared a new position paper advocating the use of IQRs to calculate preliminary TRRs as the new standard (Hart et al. 2025). Using this method, initial results on serum concentrations in children and adolescents were published as part of a large naturalistic prospective multicenter therapeutic drug monitoring/ pharmacovigilance trial (TDM- VIGIL) proposing preliminary therapeutic ranges for sertraline (Tini et al. 2022), fluvoxamine (Taurines et al. 2024), fluoxetine (Frey et al. 2023), and aripiprazole (Riegger et al. 2025). However, there is still a need for further studies to evaluate TRRs of atypical antipsychotics and SSRIs in children and adolescents.

The objective of this study is to retrospectively evaluate the serum concentrations of SSRIs and atypical antipsychotics in children and adolescents in order to provide guidance for the definition of TRRs in this age group and thus contribute to making drug therapy safer and more effective.

## Materials and methods

### Study design and study population

In this retrospective study, the clinical and TDM data of patients treated with four SSRIs (citalopram, fluoxetine, fluvoxamine and sertraline) and five atypical antipsychotics (aripiprazole, clozapine, olanzapine, quetiapine and risperidone), collected as part of routine care at the Department of Child and Adolescent Psychiatry, Psychotherapy and Psychosomatics at the University Medical Center Freiburg, were analyzed. Patients between 6 and 21 years with a valid TDM measurement of one of the named substances were included regardless of the patient’s diagnosis or treatment setting (inpatient or outpatient). Patients were excluded if the serum concentration of the drug was not detectable, information was missing in the documentation or whenever a note regarding non-adherence or medication pause was found in the medical records. Age, gender, setting, date of blood withdrawal, the presence or absence of comedication, and serum concentrations were recorded. Due to psychiatric comedication, the same patients were sometimes represented with several drugs investigated in this study. Information such as diagnosis, weight, dose, treatment response, and adverse effects were not available in all patients and not taken into account.

The survey period varied for each drug, but overall, it covered the years 1999 to 2022. The quality criteria for TDM-studies were applied according to Ulrich et al. (1998) and Kloosterboer et al. (2020) and include a standardized analytical method, steady state conditions during blood withdrawal, representative sample selection of patients, and sample sizes of at least ten or more individuals. The study was approved by the ethics committee Freiburg with the application number: 23-1095-S1-retro.

### Selection and assessment of serum concentrations

One serum level concentration was evaluated per patient for each drug. This study focuses on serum concentrations of parent compounds of the respective drugs. The metabolite concentrations were analyzed only for aripiprazole and risperidone.

If more than one serum concentration was measured for a patient, the most recent value was taken into account, with a few exceptions explained as follows: If a patient’s drug dose was 0 mg at the last measurement among multiple measurements, the last reading with a dose not equal to 0 mg was considered. If the patient’s age was more than 21 years at the last measured value among multiple concentrations, the last measured value at which the patient’s age was still below 21 years was taken into account.

In addition to the serum concentrations of the parent compound, the metabolite concentrations of aripiprazole (dehydroaripiprazole) and risperidone (9-hydroxyrisperidone) were analyzed. The metabolites norsertraline, desmethylclozapine, and desmethylolanzapine were not analyzed. No metabolites were collected for fluoxetine, quetiapine, fluvoxamine and citalopram.

Serum levels were measured by HPLC-UV detection or HPLC-tandem MS. For the HPLC-UV detection, standard solutions und controls were obtained from Chromsystems Instruments & Chemicals GmbH, Germany. For the HPLC-tandem, MS detection quantification was performed using standard solutions from RECIPE, Germany. For both methods, external control samples were analyzed quarterly, LGC Standard, Germany.

### Statistical analyses

The statistical analysis and the graphs were created with Microsoft Excel ^®^ for Mac (Version 16.64, Microsoft, Microsoft Deutschland GmbH, Munich) and R ^®^ for macOS (Version 4.2.1, RStudio, PBC, 250 Northern Ave, Boston, MA 02210) using the packages “psych”, “fitdistrplus”, “MASS”, “survival”, “flexsurv” and “actuar”.

First, the minimum, maximum, mean, median, standard deviation, 25th and 75th percentile, skewness and kurtosis were calculated. Then, the distribution of the serum concentrations was determined graphically and mathematically. This was done using a Cullen and Frey diagram, comparing the histogram and the density function of the concentration data with the density functions of the theoretical distributions, plotting the theoretical cumulative distribution functions (CDFs) as well as the CDF of the concentration data, a Q-Q plot plotting the quantiles of the concentration data against the theoretical quantiles and a P-P plot plotting the probabilities of the concentration data against the theoretical probabilities.

The following distributions were examined: Weibull, gamma, normal, lognormal, logistic, loglogistic and exponential. The calculations were carried out using the goodness of fit criteria (Akaike information criterion, Bayesian information criterion) as well as the goodness of fit statistics (Kolmogorov- Smirnov statistics, Cramer- von Mises statistics, Anderson- Darling statistics). The graphical and mathematical analysis was used to decide which distribution the data most closely followed. The fitted distributions were primarily analyzed using the graphical methods; the mathematical methods are supportive, but they are not objective instruments for clearly validating or rejecting a fitted distribution.

The serum concentration ranges calculated by using the 25th and 75th percentiles based on the median of the data were compared to the ranges calculated by the “mean ± standard deviation-method”, the 25th−75th percentile ranges determined from the fitted distribution and the TRRs for adults published by the AGNP (Hiemke et al. 2018).

## Results

### Study population

**Fig. 1:**
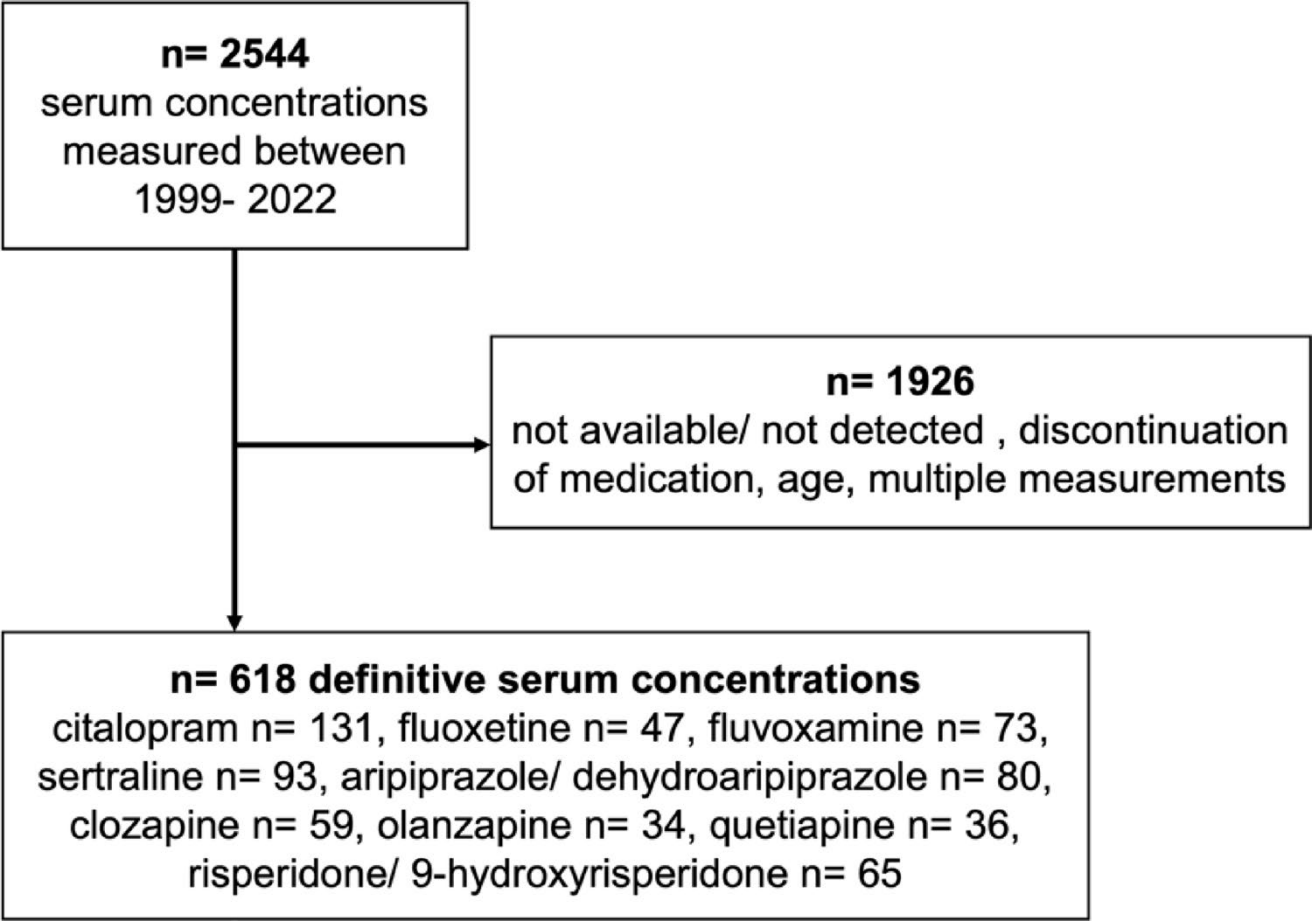
Study flow chart of serum concentrations analyzed in the study

Overall, 670 patients with in total 2544 serum concentration measurements were screened for participation in the study. 45 patients with 130 serum concentration measurements were excluded because some information in the documentation was missing or no concentration was detectable. One patient treated with fluoxetine was excluded as there were indications of discontinuation of the drug. By rechecking the age of the patients, six patients with six concentrations were excluded. As only one serum level per patient was considered, 1789 serum levels were not included in the analysis. The definitive patient collective consisted of 618 patients and serum concentrations (Fig. 1).

Comedication was documented in 103 out of 618 patients; only the medication was recorded and not its dose.

Overall, 66% of the patient population was female and 34% male. The age range (in years) of the patient population for the individual drugs was: citalopram: 15.3–17.7, fluoxetine: 15.2–18.2, fluvoxamine: 13.4–18.0, sertraline: 15.0-17.6, aripiprazole/ dehydroaripiprazole: 13–17.6, clozapine: 16.0-19.6, olanzapine: 14.1–18.1, quetiapine: 15.8–18.0 and risperidone/ 9-hydroxyrisperidone: 13.0–18.0. 85% were in-patients and 15% were out-patients (Table 1).

**Table 1:** Definitive patient collective

Drug	Number of patients	Setting (inpatients/outpatients)	Sex (% males)	Age in years (mean ± SD)
All	618	525/96	34	16.2 ± 1.9
Citalopram	131	116/15	20	16.5 ± 1.2
Fluoxetine	47	37/10	26	16.7 ± 1.5
Fluvoxamine	73	43/30	36	15.7 ± 2.3
Sertraline	93	90/3	16	16.3 ± 1.3
Aripiprazole/dehydroaripiprazole	80	72/8	41	15.3 ± 2.3
Clozapine	59	45/14	58	17.8 ± 1.8
Olanzapine	34	31/3	38	16.1 ± 2.0
Quetiapine	36	33/3	33	16.9 ± 1.1
Risperidone/9-hydroxyrisperidone	65	55/10	60	15.5 ± 2.5

*SD* standard deviation

### Probability distributions and interquartile ranges of collected serum concentrations

**Fig. 2:**
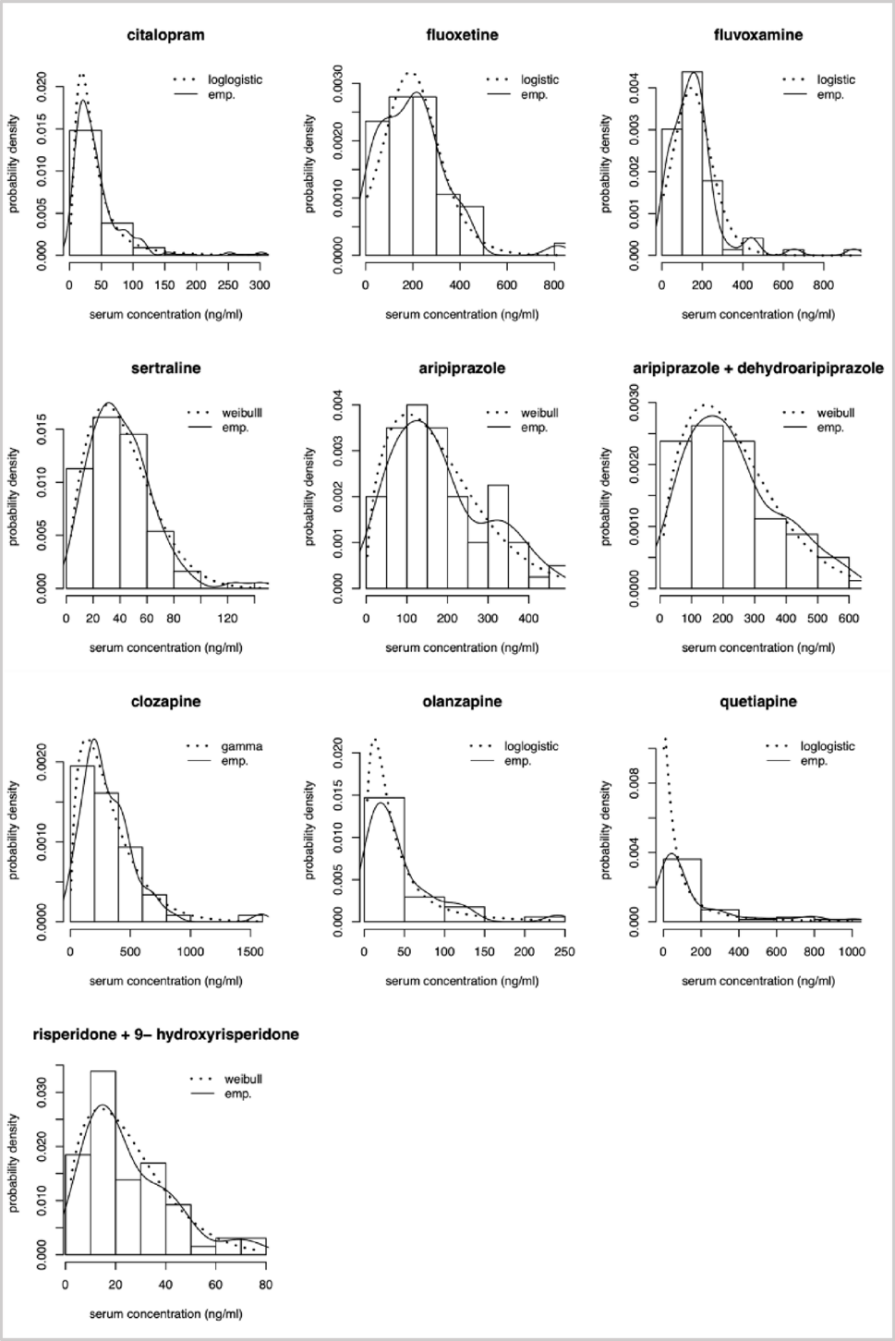
Presentation of the probability density functions (PDFs) of the distributions with the best fit in comparison with the density functions (labeled as “emp.” in the figure) and the histograms of the serum concentrations of citalopram, fluoxetine, fluvoxamine, sertraline, aripiprazole, aripiprazole + dehydroaripiprazole, clozapine, olanzapine, quetiapine and risperidone + 9- hydroxyrisperidone. *emp.* empiricial

The distribution of the data was determined by graphical analysis. For citalopram, quetiapine and olanzapine, the data most closely followed a loglogistic distribution. For aripiprazole, aripiprazole plus dehydroaripiprazole, sertraline and risperidone plus 9-hydroxyrisperidone, the weibull distribution was most fitting. The data for fluoxetine and fluvoxamine followed a logistic distribution, whereas the data for clozapine clearly followed a gamma distribution. See Fig. 2 for the presentation of the distributions in comparison with the density functions and the histograms of the concentration data.

Due to the small number of study participants, it was not possible to make a clear allocation as to which distribution the data followed in the majority of cases. For some drugs, the graphical evaluation was consistent with the mathematical tests and clearly indicated a specific distribution. This was the case for citalopram (loglogistic), quetiapine (loglogistic) and aripiprazole plus dehydroaripiprazole (weibull). For the other drugs, the different goodness of fit tests indicated different possible distributions and sometimes even conflicted with the graphical evaluation.

In order to provide guidance on age specific therapeutic reference ranges the IQR were calculated as follows: 18–51 ng/ml for citalopram, 102–263 ng/ml for fluoxetine, 75–203 ng/ml for fluvoxamine, 25–54 ng/ml for sertraline, 93–245 ng/ml for aripiprazole, 118–313 ng/ml for aripiprazole plus dehydroaripiprazole, 180–419 ng/ml for clozapine, 13–57 ng/ml for olanzapine, 34–234 ng/ml for quetiapine, and 11–35 ng/ml for risperidone plus 9-hydroxyrisperidone.

Table 2 shows the serum concentration ranges determined in this study using the IQR (25th to 75th percentile), the mean ± standard deviation, the IQR of the distribution with the best fit and the comparison with the therapeutic reference ranges for adults proposed by the AGNP.

The upper and lower limits of the IQRs of the serum concentrations and the IQRs of the distributions with the best fit are very similar in the case of sertraline, citalopram, olanzapine, risperidone + 9- hydroxyrisperidone, Aripiprazole and Aripiprazole plus dehydroaripiprazole. A greater deviation is seen at the upper limit for Fluoxetine and fluvoxamine. For clozapine and quetiapine, both the lower and upper limits of the IQRs of the distribution with the best fit deviate more strongly from the IQRs of the concentration data. The more similar these values are, the better is the fit of the distribution. The greater the deviation is, the worse the fit. In such cases, the distribution of the data is not scalable to clinical populations, but only serves to help select the appropriate calculation method for the TRR.

**Table 2:** Serum concentration ranges for children and adolescents in ng/ml calculated by the interquartile range (25th to 75th percentile), compared to the IQR of the fitted distributions, the mean ± SD and to the therapeutic ranges from the AGNP defined for adults (Hiemke et al. 2018)

Drug	Suggested IQR	IQR fitted distribution	Mean ± SD ng/ml	TRR for adults
Citalopram	18–51	Loglogistic: 18–51	1–84	50–110
Fluoxetine	102–263	Logistic: 104–275	51–350	–
Fluoxetine + N-desmethyl fluoxetine	–	–	–	120–500
Fluvoxamine	75–203	Logistic: 78–215	21–310	60–230
Sertraline	25–54	Weibull: 23–56	17–66	10–150
Aripiprazole	93–245	Weibull: 89–245	63–294	100–350
Aripiprazole + dehydroaripiprazole	118–313	Weibull: 121–317	86–378	150–500
Clozapine	180–419	Gamma: 141–429	66–566	350–600
Olanzapine	13–57	Loglogistic: 15–54	– 4–94	20–80
Quetiapine	34–234	Loglogistic: 25–174	– 80–428	100–500
Risperidone + 9-hydroxyrisperidone	11–35	Weibull: 12–34	8–42	20–60

*IQR* interquartile range, *SD* standard deviation, *TRR* therapeutic reference range according to the consensus guidelines for TDM of the AGNP

**Fig. 3:**
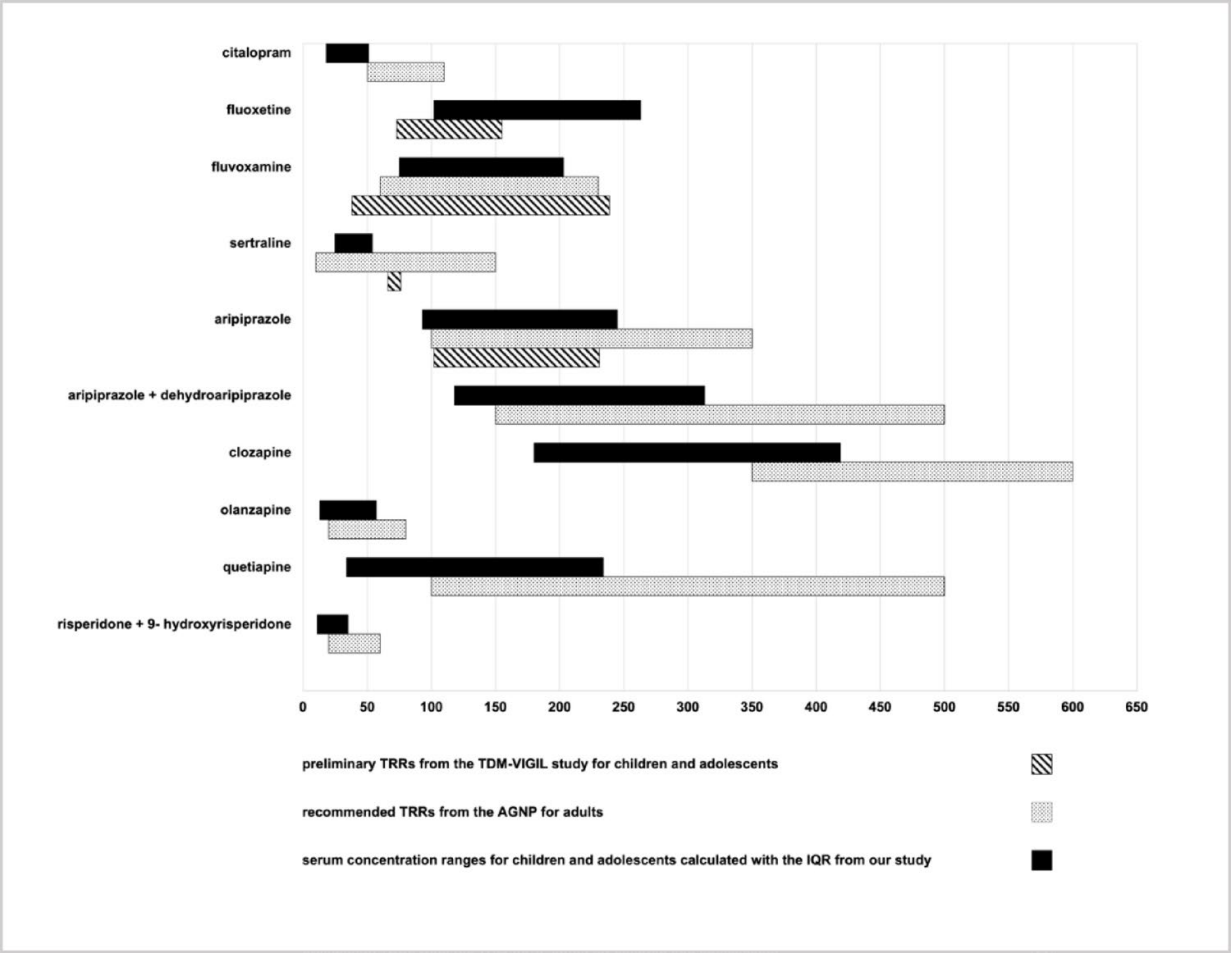
Serum concentration ranges for children and adolescents in ng/ml calculated by the interquartile range (25th−75th percentile) compared to the therapeutic reference ranges from the AGNP (Hiemke et al. 2018) for adults if available and the results of the TDM VIGIL study for fluoxetine (Frey et al. 2023), fluvoxamine (Taurines et al. 2024), sertraline (Tini et al. 2022), and aripiprazole (Riegger et al. 2025). *IQR* interquartile range, *AGNP* Arbeitsgemeinschaft für Neuropsychopharmakologie und Pharmakopsychiatrie, *TDM-VIGIL* Therapeutic Drug Monitoring-Pharmacovigilance study

See Fig. 3 for a visual comparison of our IQR ranges with the suggested reference ranges of the multicenter TDM- VIGIL study (fluoxetine, fluvoxamine, sertraline and aripiprazole ) and the AGNP.

## Discussion

We analyzed serum concentrations of four SSRIs and five atypical antipsychotics in children and adolescents in order to provide guidance for the definition of TRRs in this age group. Because the data was not normally distributed in all drugs, the calculation of the IQR as a basis for a preliminary range is suggested.

A recent pediatric TDM study on sertraline calculated a higher and narrower range of 66–76 ng/ml based on 14 subjects with OCD who responded well to the treatment (Tini et al. 2022). A direct comparison is not possible, sertraline often is applied in higher doses in patients with OCD.

In our study, only the parent substance fluoxetine was investigated but not the active metabolite norfluoxetine. Nevertheless, our results can be used for comparison with other studies that also investigated the serum concentration of the parent substance as part of their study, such as Frey et al. (2023) and Reis et al. (2009). Frey et al. (2023) proposed a transdiagnostic TRR of 73 to 155 ng/ml for fluoxetine, the lower limit of which is similar with our case, but our upper limit is much higher. One possible reason could be that in our study, in contrast to Frey et al. (2023), no distinction was made between responders and non-responders when calculating the IQR, which according to Hart et al. (2025) can lead to a broader range.

The IQR range determined from our trans-diagnostic data for risperidone plus 9-hydroxyrisperidone (11–35 ng/ml) is similar to other studies. Klampfl et al. (2010) recommended 8–26 ng/ml for impulsive-aggressive behavior, Kloosterboer et al. (2021) advised 15–25 ng/ml for autism spectrum disorders (ASD) and Taurines et al. (2022) proposed 9–33 ng/ml for schizophrenia. Conversely, another study by Hermans et al. (2024) suggested a much lower range of 3.5–7 ng/ml for ASD.

For aripiprazole, a transdiagnostic reference range in minors of 61–327 ng/ml and 106–375 ng/ml for schizophrenia was determined, with both upper limits much higher than ours (Egberts et al. 2020). In another recently published study for aripiprazole, the proposed ranges for pediatric patients were 102–231 ng/ml for schizophrenia - spectrum disorder, 62–159 ng/ml for affective disorders and 63–143 ng/ml for behavioral, emotional or tic disorders. The proposed reference range for aripiprazole in the treatment of schizophrenia is similar to our results (Riegger et al. 2025). An important limitation of both of this studies is that the concentrations of the metabolite dehydroaripiprazole were not measured (Egberts et al. 2020; Riegger et al. 2025). According to Taurines et al. (2024), a preliminary age specific TRR of 55–371 ng/ml was determined for fluvoxamine for OCD; the transdiagnostic range was 38–239 ng/ml. Our calculated transdiagnostic IQR range for fluvoxamine is narrower overall. A clearly defined TRR for quetiapine for minors does not yet exist, but in one study 67.8% of the serum level concentrations were below the lower limit (100 ng/ml) of the TRR recommended for adults (Grimm al. 2006; Albantakis et al. 2017). This supposedly lower limit in minors is also reflected in our study. A 2017 study shows that the vast majority of serum level concentrations of olanzapine are within the recommended TRR for adults, and concluded a similar TRR for adults and minors (Hiemke al. 2002; Fekete et al. 2017). Our IQR range calculated for olanzapine also leads to this conclusion, as it is relatively similar to that defined by the AGNP for adults (Hiemke et al. 2018). A more recent paper even suggests a TRR of 20–40 mg/nl for adults which is even more similar to ours (Wesner et al. 2023). In the case of clozapine, there is a study in which more than half of the serum concentrations in children and adolescents were below the suggested range for adults, suggesting a lower TRR overall (Koponenet al. 1996; Wohkittel et al. 2016). Concluding from our data, we also suggest a significantly lower TRR for the use of clozapine in minors versus adults. For citalopram and aripiprazole plus dehydroaripiprazole, no study could be found in which a TRR for children and adolescents was specifically determined or recommended. Only a small proportion of the reference ranges published by the AGNP (Table 2) are derived from randomized clinical trials; they are mainly derived from studies with therapeutically effective doses. In comparison, for sertraline, aripiprazole and aripiprazole plus dehydroaripiprazole the lower limit of the range is similar to our calculations, but the upper limit is much higher. The proposed range for fluvoxamine, olanzapine and risperidone plus 9- hydroxyrisperidone is the most similar to the IQR ranges in our study (Brøsen et al. 1993). In contrast, for clozapine and quetiapine, both the lower and upper recommended limits are significantly higher than the ranges derived from our data. Possible explanation for our lower values could be due to lower doses in polypharmacy or enzyme inhibiting.

For citalopram, the preliminary TRR which could be derived from our data is almost entirely below the recommended lower limit of the AGNP.

This massive deviation for citalopram from the AGNP range for adults cannot be fully explained. There are no comparative studies in children and adolescents with which IQR range could be compared for plausibility. In adults, it was found that an occupancy of the serotonin transporter 5-HTT of 80% is necessary for maximum clinical effectiveness, which corresponds to a serum concentration of at least 50 ng/ml (Ostad Haji et al. 2011).

The comparative data for fluoxetine was insufficient, since the reference range calculated by the AGNP is composed of the parent substance and the active metabolite (Hiemke et al. 2018).

Flexible dosing regimens and the exclusion of concentrations that are in the subtherapeutic or lower range can negatively influence the relationship between efficacy and antidepressant concentration (Funk et al. 2022). Flexible dose studies and TDM databases are not suitable for showing a correlation between concentration and effect. However, they can indeed be used to determine the drug concentration in the blood of responders (Hiemke 2019). Established TRRs for some antipsychotics have been verified with positron emission tomography (PET) studies. In the case of haloperidol, risperidone and olanzapine, it is recommended to lower the upper limit of the reference range to reduce the risk of extrapyramidal side effects (EPS), as the PET results only support the lower limit. For aripiprazole and clozapine, the optimal receptor occupancy is consistent with the proposed reference ranges (Hart et al. 2024b). Such an investigation was also carried out for SSRIs. With the exception of fluvoxamine, which already exhibits high sertraline transporter (SERT) binding at levels three times below the recommended reference range, receptor occupancy is consistent with the recommended reference ranges for the SSRIs (Hart et al. 2024a).

However, these PET studies refer to adults; no PET studies exist for children and adolescents.

Both a retrospective study (Kumar et al. 2016) as well as a meta-analyses have shown that lower doses of antipsychotics may be sufficient in the maintenance treatment of schizophrenia than in the acute phase (Uchida et al. 2011). This raises the question of whether this should be taken into account when establishing the TRR. The administration of more than one antidepressant or atypical antipsychotic is widespread. In a study of 2177 patients with schizophrenia, 42.9% of them received two or more antipsychotics, while 25.4% of the 1169 patients with major depressive disorder received two or more antidepressants (Hashimoto et al. 2021). The average doses of antipsychotics in monotherapy are usually higher than in polypharmacy (Faries et al. 2005). In our study, the measured serum levels for the individual substances could be lower in cases of polypharmacy than with monotherapy, and thereby distort the reference range.

### Limitations

This study has some limitations that must be taken into account when evaluating the results. First, the number of serum concentrations evaluated per drug is sometimes quite low. Due to the small sample size, the distribution of the data cannot be applied to clinical populations. Secondly, we could not guarantee steady-state conditions in this sample. Thirdly, this study focused exclusively on serum concentrations. Possible distortions of the results that could occur due to concomitant medication, diagnosis, dosage, gender, weight or other factors were not taken into account. In particular, it should be pointed out that comedication with psychotropic drugs, which some of our patients have been receiving, can lead to drug-drug interactions. And fourthly, there is a lack of pharmacodynamic data on clinical efficacy and tolerability. The amalgamation of data from responders with nonresponders leads to a broader therapeutic range than that in responders only. The advantage, however, is that a broader range can contribute to better comparability with other laboratories where there is also little information on clinical effects available.

## Conclusion

Although the sample size was relatively small, the dosage and therapeutic effects were not taken into account our data provide valuable information on serum concentrations in children and adolescents and can contribute to the definition of age specific TRRs. TRRs in this age group should be further investigated in studies with larger sample sizes and fixed dosages. It would be useful to establish TRRs for different diagnoses.
